# Volatile Organic Compounds Produced by Co-Culture of *Burkholderia vietnamiensis* B418 with *Trichoderma harzianum* T11-W Exhibits Improved Antagonistic Activities against Fungal Phytopathogens

**DOI:** 10.3390/ijms252011097

**Published:** 2024-10-16

**Authors:** Wenzhe Li, Xinyue Wang, Yanqing Jiang, Shuning Cui, Jindong Hu, Yanli Wei, Jishun Li, Yuanzheng Wu

**Affiliations:** 1 Shandong Provincial Key Laboratory of Applied Microbiology, Ecology Institute, Qilu University of Technology (Shandong Academy of Sciences), Jinan 250103, China; lwenzhe1008@163.com (W.L.); wangxinyue9908@163.com (X.W.); yanqing597976@163.com (Y.J.); 13335151750@163.com (S.C.); hujd@sdas.org (J.H.); yanli_wei@163.com (Y.W.); yewu2@sdas.org (J.L.); 2 School of Bioengineering, Qilu University of Technology (Shandong Academy of Sciences), Jinan 250353, China

**Keywords:** volatile organic compounds (VOCs), metabolomic profiles, co-culture, *Burkholderia vietnamiensis*, *Trichoderma harzianum*, antagonistic activity, *Botrytis cinerea*, *Fusarium oxysporum*

## Abstract

Recently, there has been a growing interest in the biocontrol activity of volatile organic compounds (VOCs) produced by microorganisms. This study specifically focuses on the effects of VOCs produced by the co-culture of *Burkholderia vietnamiensis* B418 and *Trichoderma harzianum* T11-W for the control of two phytopathogenic fungi, *Botrytis cinerea* and *Fusarium oxysporum* f. sp. *cucumerium* Owen. The antagonistic activity of VOCs released in mono- and co-culture modes was evaluated by inhibition assays on a Petri dish and in detached fruit experiments, with the co-culture demonstrating significantly higher inhibitory effects on the phytopathogens on both the plates and fruits compared with the mono-cultures. Metabolomic profiles of VOCs were conducted using the solid–liquid microextraction technique, revealing 341 compounds with significant changes in their production during the co-culture. Among these compounds, linalool, dimethyl trisulfide, dimethyl disulfide, geranylacetone, 2-phenylethanol, and acetophenone were identified as having strong antagonistic activity through a standard inhibition assay. These key compounds were found to be related to the improved inhibitory effect of the B418 and T11-W co-culture. Overall, the results suggest that VOCs produced by the co-culture of *B. vietnamiensis* B418 and *T. harzianum* T11-W possess great potential in biological control.

## 1. Introduction

In recent years, increasing attention has been paid to the biocontrol activities of volatile organic compounds (VOCs) secreted by microorganisms [1]. VOCs are small molecular compounds with low molecular weights (averaging less than 300 Da), lipophilic properties, high vapor pressure, and low boiling points [2,3,4]. Due to their physico-chemical properties, VOCs can effortlessly diffuse through pores filled with gas and water. Consequently, VOCs can be utilized as signaling molecules for long- or short-distance travel [5]. Compared with non-volatile antimicrobial substances, VOCs are more readily adsorbed by plants in the form of chemical signals, thereby fulfilling their antimicrobial function [6]. It has been suggested that VOCs may play an important role in the biocontrol of plant pathogenic oomycetes, fungi, and bacteria through antimicrobial activity [7], modulation of the host’s immune mechanisms [8], and participation in or disruption of pathogen metabolism [9]. Their biological effects, reduction in pesticide residues, and ease of application in various agricultural systems render the utilization of VOCs a promising and sustainable alternative to synthetic fungicides for the control of plant pathogens [10,11]. In recent years, in-depth investigations have been carried out in this field to offer a more comprehensive understanding of the antimicrobial mechanisms of VOCs [12]. These biologically active VOCs, such as fatty acid-derived molecules, terpenoids, phenolic compounds, sulfides, and benzene compounds, have been demonstrated to have substantial inhibitory effects on plant pathogens [13,14].

*Burkholderia* spp. possess considerable biocontrol potential and have been demonstrated to generate VOCs that facilitate plant growth and augment plant resistance to both biotic and abiotic stresses [15,16,17]. It has been reported that *Burkholderia gladioli* E39CS3 and *B. pyrrocinia* mHSR5 showed outstanding antagonistic activity against significant phytopathogens such as *Fusarium oxysporum* and *Rhizoctonia solani* [18,19]. Additionally, the VOCs generated by *Burkholderia* spp. inhibit the mycelial cells of *Botrytis cinerea* in tomato fruits and prevent the mycelial development and spore germination of *B. cinerea* [20]. Research indicates that dimethyl disulfide (DMDS) released by *Burkholderia* spp. is a critical volatile compound implicated in the mortality of *Meloidogyne* spp. [21] and that it inhibits the growth and development of *Meloidogyne* spp. in the soil, mainly through infiltration into the soil [22].

*Trichoderma* spp. has been investigated more as an internationally recognized microorganism for the control of plant soil-borne diseases and the remediation of the soil environment [23]. Its mechanism of action encompasses competitive effects [24], hyperparasitism [25], cooperative effects or antagonistic effects [26], and induced resistance [27] and is the important reason for the extensive application and remarkable success of *Trichoderma* spp. For instance, the VOCs released by *T. virens* G-41, *T. harzianum* T-22, and *T. asperellum* ICC 012 can significantly inhibit the growth of *F. oxysporum*, *F. moniliforme*, and *B. cinerea* [28,29]. It has also been demonstrated that VOCs released by *T. virens* TRS 106 can induce a plant defense response, thereby enhancing their resistance to *B. cinerea* [30]. In addition, *Trichoderma* VOCs have direct or indirect biological control mechanisms against *Meloidogyne* spp., which is also one of the reasons why the VOCs of *Trichoderma* spp. are a kind of nematode disease control fungus with great biocontrol potential [31].

Co-cultivation has emerged as a key technology for activating latent biosynthetic pathways and uncovering novel antimicrobial metabolites [32]. It has been discovered that three communication modalities might exist when microbes coexist in the same environment: (i) One strain releases small molecules that induce the production of new metabolites by the other; (ii) exogenous molecules serve as chemical defenses to generate antibiotics or competing signaling molecules; and (iii) cell-to-cell contact initiates the silencing of clusters of genes to regulate biosynthetic enzymes [33,34]. This also raises great interest in comprehending the role of microbial co-culture in inducing the production of novel chemicals.

Our laboratory previously screened two strains with excellent biocontrol potential: *Burkholderia vietnamiensis* B418 [35] and *Trichoderma harzianum* T11-W [36]. The study aims to investigate the differences in VOCs and production among the mono- and co-culture modes of B418 and T11-W, as well as their impact on the prevention and control of pathogenic fungi. The findings will provide a foundation for the development and utilization of composite microbial pesticides.

## 2. Results

### 2.1. Morphological Characteristics of B418 and T11-W in Mono- and Co-Culture Modes

**Figure 1:**
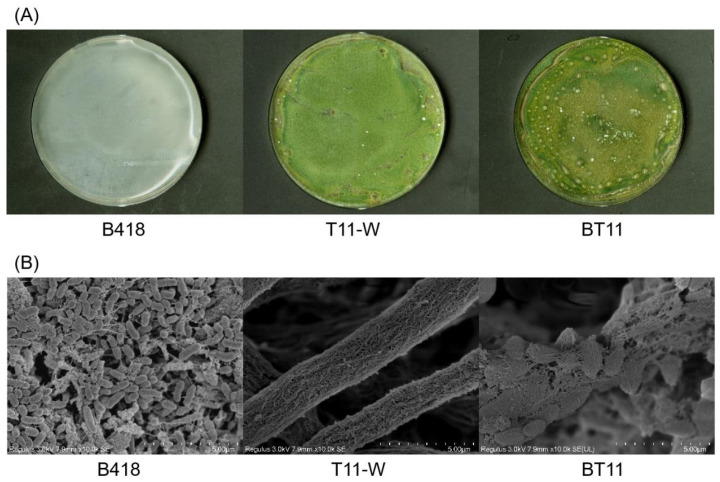
Growth morphology of *Burkholderia vietnamiensis* B418 and *Trichoderma harzianum* T11-W in mono- and co-culture modes on PDA plates (**A**) and scanning electron microscopy (SEM) of mycelium and bacterial morphology (**B**). Size bars for SEM: 5 μm. B418, *B. vietnamiensis* B418 mono-culture; T11-W, *T. harizanum* T11-W mono-culture; BT11, B418+T11-W co-culture.

In comparison with the mono-culture, the surface of the *Trichoderma* filaments appeared roughened and adhered to the B418 biofilm in the co-culture mode. Additionally, they were firmly bound to the *Trichoderma* filaments, which exhibited a tightly ordered structure (Figure 1A,B). These findings suggest that B418 and T11-W can coexist within the co-culture mode, creating a conducive environment for microbial growth and facilitating the production of secondary metabolites.

### 2.2. Antagonistic Activity of VOCs in Mono- and Co-Cultures

**Figure 2:**
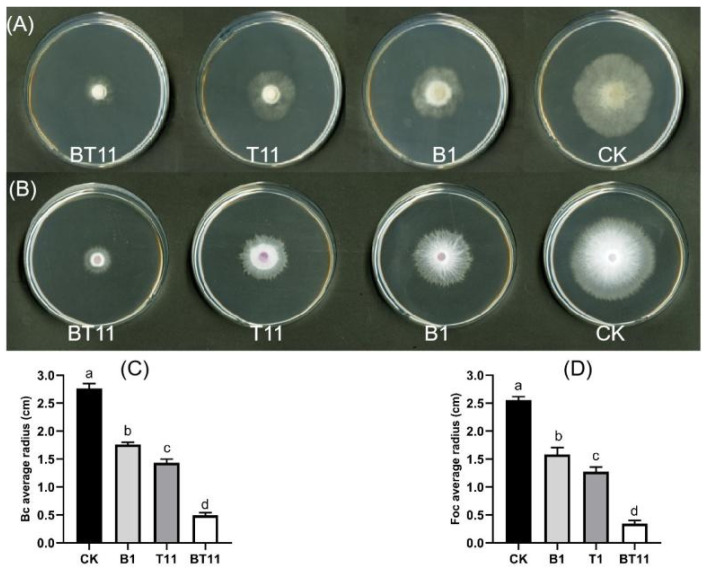
Inhibition activity of VOCs on mycelial growth of *Botrytis cinerea* (**A**), and *Fusarium oxysporum* f. sp. *cucumerium* Owen (**B**); the radius of Bc colony (**C**), and Foc colony (**D**). Error bars represent standard errors, the letters (a to d) above columns represent the significant differences at *p* < 0.05 according to one-way ANOVA with Duncan’s multiple range test. CK, control of sterile distilled water; B1, *B. vietnamiensis* B418 mono-culture; T11, *T. harizanum* T11-W mono-culture; BT11, B418+T11-W co-culture.

The inhibitory effects of VOCs produced by B418 and T11-W in both the mono-culture and co-culture modes on the mycelial growth of phytopathogens were determined using the mycelial growth rate method. The results indicated that the inhibitory effect of VOCs produced by co-culturing T11-W with B418 on phytopathogens was significantly higher than that observed in the mono-cultures (Figure 2). In the co-culture mode, the VOCs produced were able to inhibit the growth of the mycelium of the phytopathogens and caused it to exhibit irregular growth. Among them, the VOCs produced in the B418+T11-W co-culture exhibited an 82.21% inhibition against Bc. In comparison with the B418 and T11-W mono-cultures, the inhibition rates were enhanced by 45.72% and 33.9%, respectively. Furthermore, the inhibition rate against Foc reached 86.57%, which was increased by 48.5% and 36.21%, respectively, compared with the mono-culture modes. It is hypothesized that the co-culture mode may lead to the production of more, or even novel, antimicrobial substances, thereby enhancing the antagonistic effect.

### 2.3. Validation of the Antagonistic Effect of VOCs Produced by Mono- and Co-Cultures

**Figure 3:**
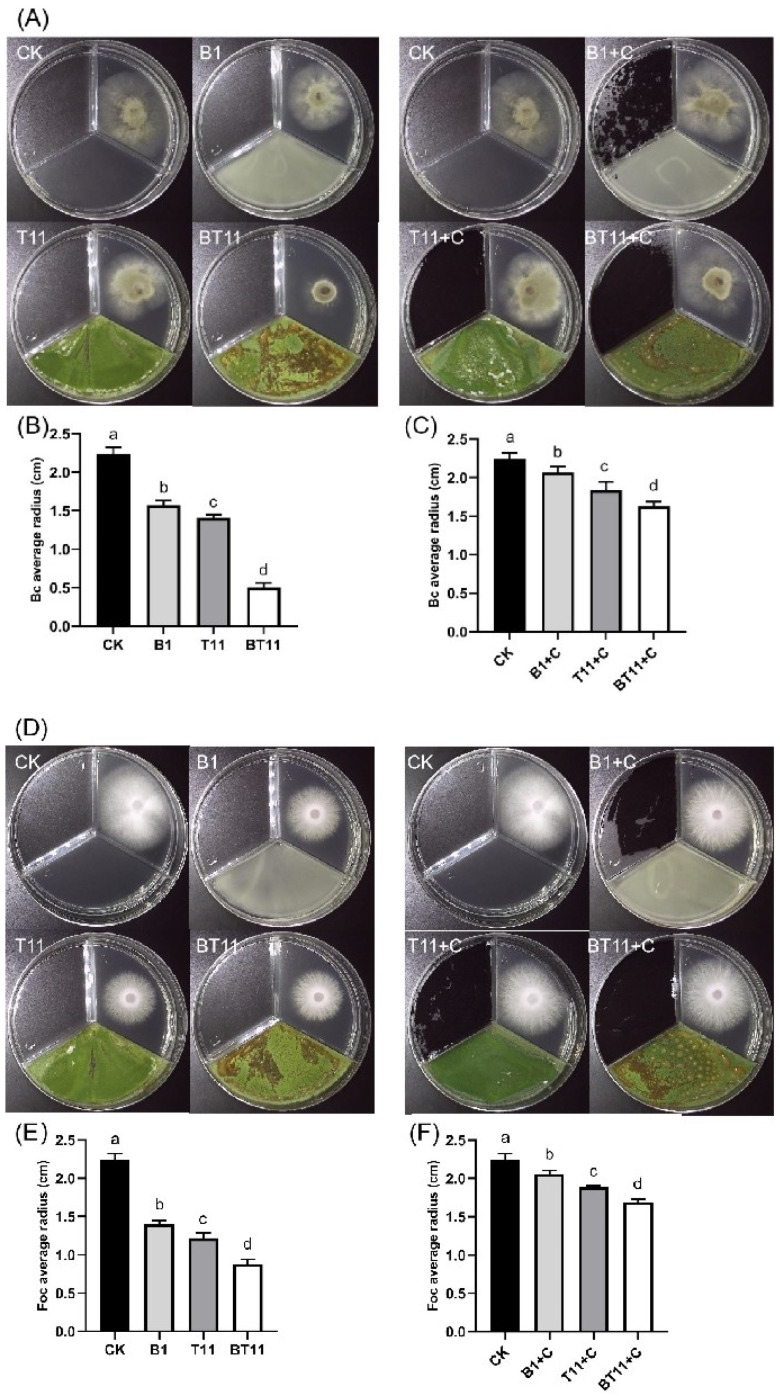
The antagonistic effect of VOCs on the mycelial growth of *B. cinerea* (Bc) and *F. oxysporum* f. sp. *cucumerium* Owen (Foc) in the three-grid determination with and without activated carbon. (**A**) The inhibitory activity of VOCs on the mycelial growth of Bc with and without activated carbon; (**B**) radius of Bc without activated carbon; (**C**) radius of Bc with activated carbon; (**D**) the inhibitory activity of VOCs on the mycelial growth of Foc with and without activated carbon; (**E**) radius of Foc without activated carbon; (**F**) radius of Foc with activated carbon. Error bars represent standard errors; the letters (a to d) above columns represent the significant differences at *p* < 0.05 according to one-way ANOVA with Duncan’s multiple range test. CK, control of sterile distilled water; B1, *B. vietnamiensis* B418 mono-culture; T11, *T. harizanum* T11-W mono-culture; BT11, B418+T11-W co-culture; C, activated carbon.

The antagonistic effect of the VOCs produced by the mono- and co-cultures was verified using the three-compartment plate method (Figure 3). The results indicated that the B418 mono-culture inhibited Bc and Foc by 22.17% and 29.27%, respectively, compared to the treatment group with the addition of activated charcoal. Additionally, the T11-W mono-culture inhibited Bc and Foc by an additional 18.9% and 29.72%, respectively; while the B418+T11-W co-culture increased to 50.3% and 36.26%, respectively. The results suggest that activated carbon adsorbs the VOCs produced by the strains, thus reducing their inhibitory effect on pathogenic fungi.

### 2.4. Verification of Inhibitory Activity on Detached Fruits

**Figure 4:**
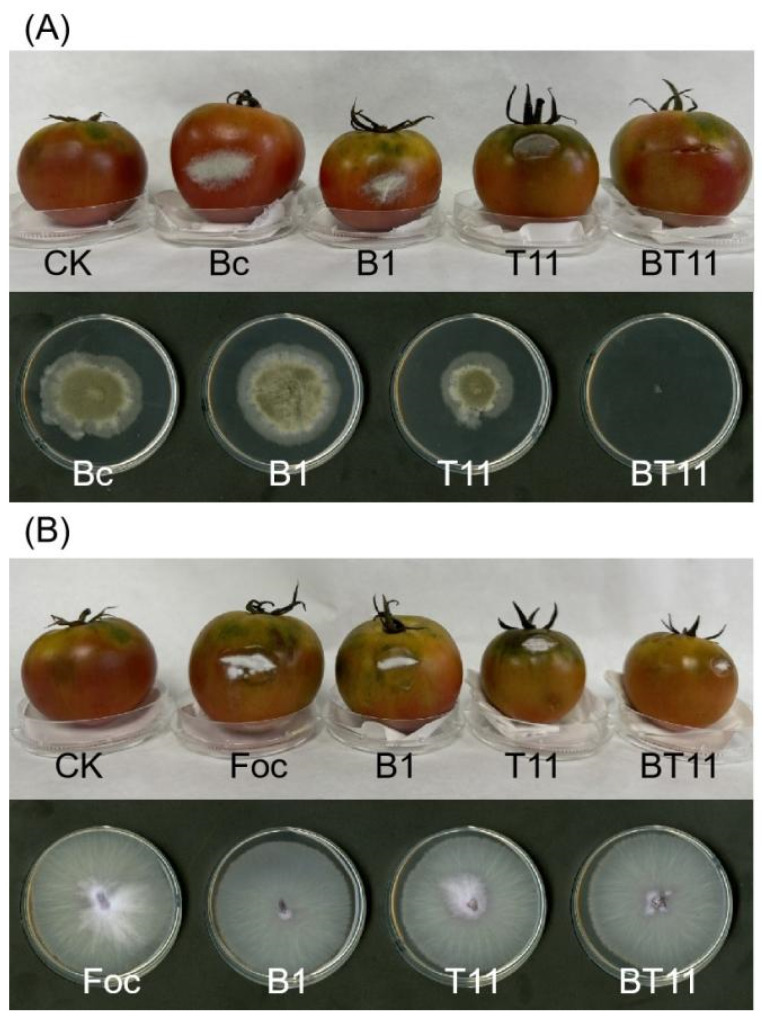
Antagonistic activity of VOCs against *Botrytis cinerea* (Bc) on tomatoes (**A**) and *Fusarium oxysporum* f. sp. *cucumerium* Owen (Foc) on tomatoes (**B**). CK, control of sterile distilled water; B1, *B. vietnamiensis* B418 mono-culture; T11, *T. harizanum* T11-W mono-culture; BT11, B418+T11-W co-culture.

The inhibitory activity of VOCs against Bc and Foc was further evaluated in detached fruit inoculation tests (Figure 4). Control tomato fruits inoculated with Bc and Foc started to develop symptoms 1 d later, characterized by white spots with sunken flesh; the spots then expanded, and by 3 d, they appeared grey and white with a rotting odor, respectively. Most of the tomato fruits treated with B418 and T11-W inoculated with Bc and Foc, respectively, exhibited more severe disease symptoms. Mycelium growth was more vigorous, while fruit rot occurred, while those treated with B418+T11-W co-cultures showed less severe disease symptoms. Remarkably, the tomato fruits inoculated with Bc did not develop any symptoms with the B418+T11-W co-culture treatment. The results of the experiment on rejoining pathogenic fungi showed a significant reduction in the activity of Bc fumigated by the T11-W and B418+T11-W co-culture. In contrast, the tomato fruit inoculated with Foc had slow mycelial growth and mild disease symptoms. Additionally, the mycelium of the pathogenic fungi treated with the B418+T11-W co-culture fumigation was unable to continue growing, while the growth and development of the Foc mycelium were not affected. Thus, the B418+T11-W co-culture can effectively inhibit the invasion and expansion of Bc and Foc on tomato fruits.

### 2.5. GC-MS Analysis of VOCs

**Figure 5:**
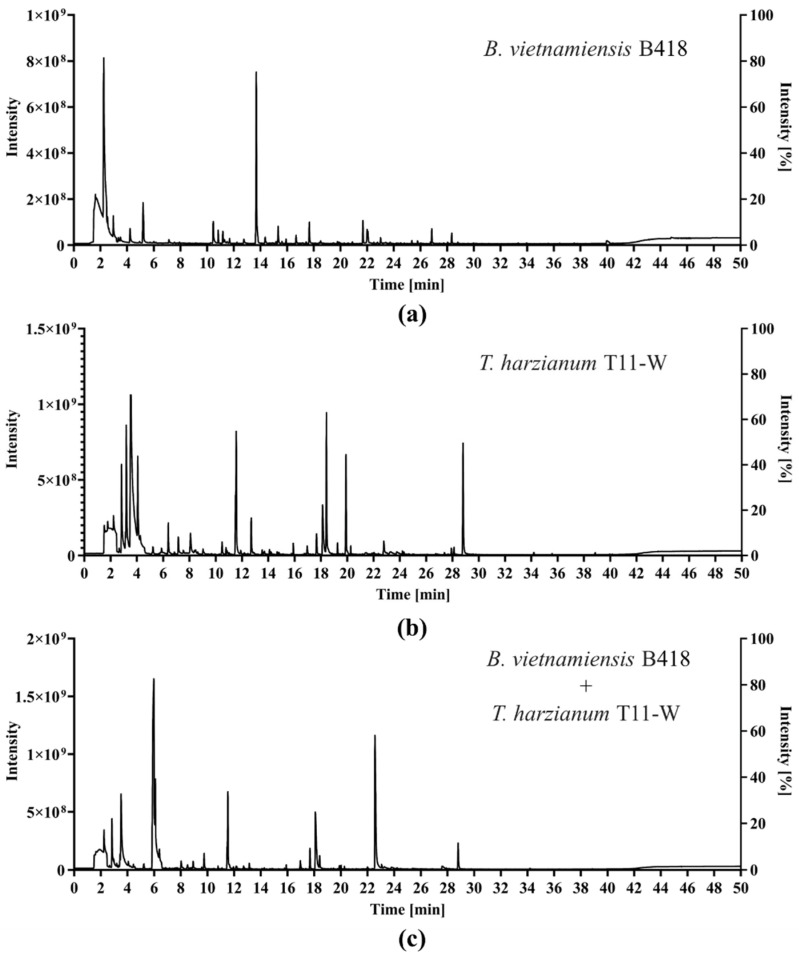
GC-MS chromatograms of *B. vietnamiensis* B418 mono-culture (**a**), *T. harizanum* T11-W mono-culture (**b**), and B418+T11-W co-culture (**c**).

GC-MS analysis was conducted to investigate the changes in volatile substance production and species before and after co-culture. The volatile substances extracted from the B418 bacterial suspension, T11-W spore suspension, and T11-W+B418 co-culture were detected using the HS-SPME method. Three biological replicates of each strain were performed to ensure the accuracy of the experimental results. As shown in Figure 5, a total of 190 compounds were identified through GC-MS analysis in the VOCs of the B418+T11-W co-culture. The results showed that 82 volatile metabolites were identified in the B418+T11-W co-culture compared to the B418 and T11-W mono-cultures, including 18 esters, 15 hydrocarbons, 12 alcohols, 7 ketones, 6 terpenoids, 3 aldehydes, and 2 B vitamins, among other compounds. The findings indicated that B418 and T11-W induced alterations in the types and relative abundance of the volatiles when co-cultured, including the generation of a novel volatile compound, isopropyl butyrate, at RT 6.08427. Geranylacetone was observed in both the B418+T11-W co-culture and the B418 mono-culture, but not in the T11-W mono-culture, and is presumably produced by B418. Additionally, T11-W was induced to produce the known monoterpene linalool, a substance that was not seen in either the single bacterial culture or the fungal cultures.

### 2.6. Screening of Differential VOCs Produced by Mono- and Co-Cultures

**Figure 6:**
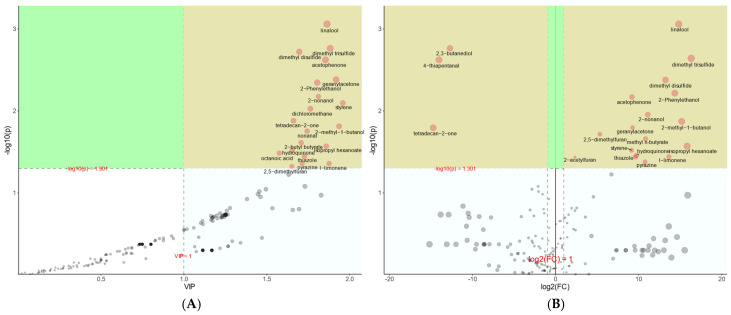
Metabolomic analysis of VOCs produced by mono- and co-cultures. Compound analysis of differences in different training models (**A**) and volcano plot analysis of VOCs (**B**).

Differential metabolite profiles of the mono-culture and co-culture groups were analyzed using a combination of VIP values greater than 1.0 and a false discovery rate (FDR) adjusted *p*-value of less than 0.05 (Figure 6A). The figure highlights the 20 differential compounds, shown in yellow, including linalool, geranylacetone, dimethyl trisulfide, 2-nonanol, dimethyl disulfide, acetophenone, methyl n-butyrate, tetradecan-2-one, dichloromethane, 2-methyl-1-butanol, nonanal, isopropyl hexanoate, hydroquinone, octanoic acid, thiazole, 2,5-dimethylfuran, 2-butyl butyrate, pyrazine, l-limonene, and 2-phenylethanol.

### 2.7. Univariate Statistical Analysis

To further evaluate the extent of the change in the differential compounds and their impact on microorganisms across different culture groups, a univariate analysis was conducted. The magnitude of change in the compounds was assessed by calculating the log_2_(FC) values [37]. In this analysis, the combinations with FC values > 2.0 and *p*-values < 0.05 were deemed to indicate significant alterations and variability in the differential metabolites. The study results revealed that 17 out of the top 20 volatile organic compounds exhibited an up-regulation trend in the co-culture mode. These compounds included linalool, dimethyl trisulfide, dimethyl disulfide, 2-phenylethanol, acetophenone, 2-nonanol, 2,5-dimethylfuran, geranylacetone, 2-methyl-1-butanol, methyl n-butyrate, isopropyl hexanoate, styrene, 2-acetylfuran, hydroquinone, thiazole, pyrazine, and l-limonene. Three additional compounds exhibited a down-regulation trend: 2,3-butanediol, 4-thiapentanal, and tetradecan-2-one (Figure 6B). Based on the results of the metabolomics data, the five VOCs with significant differences were identified as linalool, dimethyl trisulfide, dimethyl disulfide, 2-phenylethanol, and acetophenone. Furthermore, additional verification was conducted to confirm their inhibitory effects on microorganisms.

### 2.8. Effects of VOC Standards on Mycelial Growth

**Figure 7:**
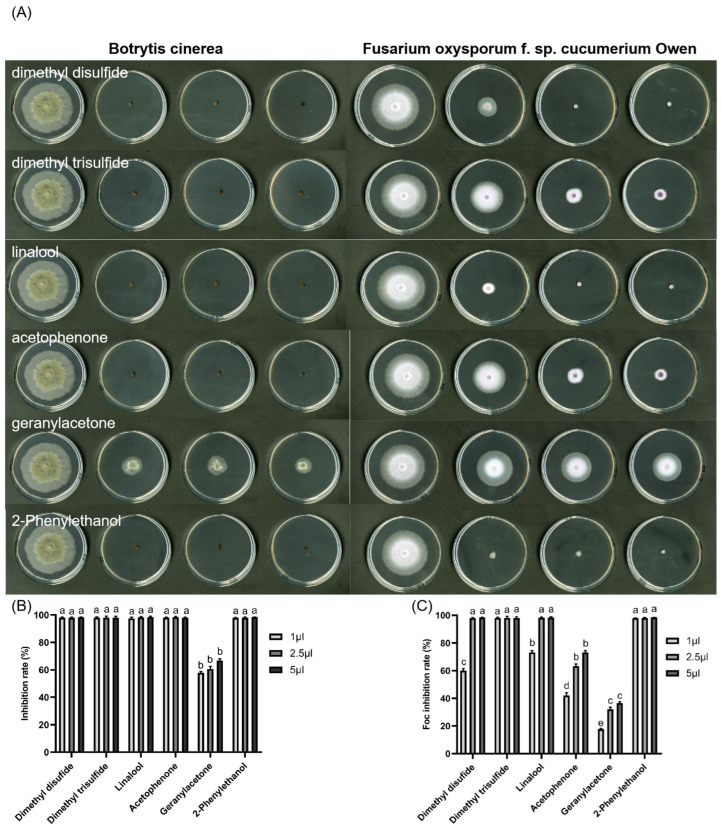
Inhibitory effect of standard VOCs on the growth of *B. cinerea* (Bc) and *F. oxysporum* f. sp. *cucumerium* Owen (Foc) on plates (**A**). In the treatments for each group from left to right were CK (0), 1, 2.5, and 5 μL of standard VOCs. The inhibition rate of standard VOCs against Bc (**B**) and Foc (**C**). Error bars represent standard errors; the letters (a to e) above columns represent the significant differences at *p* < 0.05 according to one-way ANOVA with Duncan’s multiple range test.

Based on the metabolomics data, six VOCs with significant differences were screened as linalool, dimethyl trisulfide, dimethyl disulfide, geranylacetone, 2-phenylethanol, and acetophenone. Their antagonistic effects were further validated. At a concentration of 1 × 10^−5^ mg/L, the six VOCs showed high inhibitory activities against pathogenic fungi (Figure 7). Regarding the antagonistic effect against Bc in particular, five VOCs, with the exception of geranylacetone, achieved nearly 100% inhibition. There was almost no mycelium growth observed around the nucleus. For the antagonistic effect against Foc, the results after fumigation with dimethyl trisulfide and 2-phenylethanol standards were similar to those of Bc; little mycelial growth was observed. Dimethyl trisulfide and linalool also inhibited mycelium growth near the nucleus. Dimethyl disulfide and linalool showed antagonistic activity against Foc by inhibiting its growth by 59.14% and 73.68%, respectively. It is worth noting that geranylacetone and acetophenone exhibited lower inhibitory activities against Foc; in fact, the inhibition rate of geranylacetone was even less than 20%. The experimental results indicated that the main VOCs produced by B418 and T11-W in the co-culture mode demonstrated stronger antagonistic activity against Bc.

## 3. Discussion

The volatile organic compounds (VOCs) produced by *Burkholderia* spp. have been demonstrated to enhance plant growth and improve plant resistance to both biotic and abiotic stresses [38]. These VOCs, which include terpenes and other components, are believed to play a significant role in inhibiting the growth of plant pathogenic fungi [39]. Additionally, *Trichoderma* spp., a widely used biological fungus for plant pest control, releases VOCs that can directly inhibit the growth of pathogenic fungi or induce abnormal changes in them, thus effectively suppressing plant pathogenic fungi [40].

Fungal–bacterial communities are inherently diverse and complex and exist in various ecosystems [41]. In the co-culture model, various interactions between fungi and bacteria occur, including competitive and synergistic interactions. These interactions are reflected in growth, nutrition, and reproduction. Fungal hyphae create an environment that is conducive to bacterial growth, and the bacteria attached to the hyphae can have diverse effects on the fungal host. In this study, we observed that *B. vietnamiensis* B418 could adhere to the filaments of *T. harzianum* T11-W under the co-culture mode and form a biofilm on PDA plates. Similar results were reported by Kjeldgaard et al., showing that *Bacillus subtilis* secreted a biofilm matrix on the hyphae of *Aspergillus niger* and *Agaricus bisporus* [42]. These effects include the influencing of energy dynamics as well as the production of secondary metabolites [43].

Microbial co-culture is an effective method for enhancing the production of specific VOCs by simulating microbial habitats or inducing the production of new secondary metabolites not previously observed in cultures of independent strains. This approach mimics natural microbial interactions and can lead to the discovery of novel bioactive compounds with potential industrial applications [44]. Co-culturing not only increases the species diversity of secondary metabolites but also enhances their bioactivity and production compared to culturing the strains individually. To date, co-culture studies have primarily focused on the effect of one microorganism on the metabolite profile of another. However, our current study not only demonstrates that co-culture can stimulate the production of new metabolites but also suggests that *B. vietnamiensis* B418 and *T. harzianum* T11-W may synergistically enhance the production of specific VOCs. The comparison of the chemical compositions of the pure and co-cultures revealed significant differences. In the microbial co-culture of B418 and T11-W, the bacterial strain appeared to inhibit the production of most of the fungal metabolites detected in the fungal pure culture. However, it induced the production of 82 volatile metabolites not previously detected within fungus T11-W, including 18 esters, 15 hydrocarbons, 12 alcohols, 7 ketones, 6 terpenoids, 3 aldehydes, and 2 B vitamins, among other compounds. Specifically, (1beta,7beta)-cedr-8(15)-ene, 1,5-dimethyl-1,4-cyclohexadiene, linalool, l-limonene, 1,3,6,10-cyclotetradecatetraene, and 3,7,11-trimethyl-14-(1-methylethyl)-,(s-(e,z,e)) were identified as terpenoids that did not appear in either the bacterial or the fungal mono-culture; however, geranylacetone (a terpene ketone) was present in the B418 mono-culture. It can be inferred that the expression of terpenoid chemosynthesis-related genes was promoted after the bacterial–fungal co-culture. This finding is consistent with previous research findings [45]. It is worth noting that it has not yet been observed whether the source of the corresponding precursors in other terpene chemotaxis is from the fungus itself, or due to the cell, or perhaps even from the matrix admixture, etc.

In addition to the aforementioned findings, a total of 190 compounds were identified through GC-MS analysis in the VOCs generated by the co-culture of B418 and T11-W. Subsequently, based on the metabolomics data, six VOCs with significant variations, namely linalool, dimethyl trisulfide, dimethyl disulfide, geranylacetone, 2-phenylethanol, and acetophenone, were selected for further validation of their antifungal activity. The results indicated that all six compounds exhibited effectiveness against *B. cinerea* (Bc) and *F. oxysporum* f. sp. *cucumerium* Owen (Foc). Furthermore, previous studies have confirmed the significant inhibitory effects of these VOCs on a wide range of phytopathogenic fungi. Therefore, it can be inferred that the enhanced antifungal activity following co-culture may be attributed to these organic compounds.

The present study also provides evidence that VOCs produced by the co-culture of B418 with T11-W have a significant inhibition effect on the mycelial growth and development of Bc and Foc. Specifically, in the fruit pathogen back-joining experiment, the activity of Bc fumigated by the B418+T11-W co-culture was significantly reduced, leading to the mycelium’s inability to grow on PDA plates. Furthermore, in the antifungal activity assay of volatile organic compound standards, it was observed that linalool, dimethyl trisulfide, dimethyl disulfide, geranylacetone, 2-phenylethanol, and acetophenone exhibited strong antifungal activity. However, when testing against Foc at a dilution concentration of 1 × 10^−5^ mg/L (100-fold dilution of the standard), there was a significant reduction in antifungal effect. Only dimethyl trisulfide and linalool showed effective inhibitory activity against both pathogens. It is worth noting that further investigation is needed to determine whether different compounds produce synergistic effects after fungal–bacterial co-culture to enhance inhibitory efficiency. Interestingly, it has been demonstrated that linalool resistance to tobacco mosaic virus (TMV) involves the activation of plant disease resistance through the induction of salicylic acid-mediated plant immune responses [46]. Therefore, further research is necessary to determine whether the effects observed in this study are attributable to linalool acting as a signaling molecule or can be explained solely by its antimicrobial effects.

Linalool, one of the major VOCs produced by the co-culture of B418 and T11-W, is a versatile volatile monoterpene alcohol commonly found in plants and microorganisms. This monoterpene exhibits inhibitory effects on a wide range of plant pathogens, including the inhibition of the mycelial growth of various pathogenic fungi and the germination of pathogenic fungal spores on plant surfaces [47]. Furthermore, linalool has been found to alter the structural integrity of both Gram-positive and -negative bacteria by increasing membrane permeability, leading to subsequent loss of cellular components. It was observed that *B. cinerea* infection promoted linalool production in strawberry fruit. Therefore, it is suggested that safe doses of linalool fumigation could serve as a novel and environmentally friendly biological control method for Bc. However, the potential physiological mechanisms through which linalool reduces Bc damage to tomato fruit have not been thoroughly investigated. It is widely recognized that biotic stresses, such as Bc infection, trigger an increase in reactive oxygen species (ROS) and induce oxidative stress [48]. To counteract ROS, plants have developed an antioxidant system comprising enzymatic and non-enzymatic components. Rahman et al. conducted a study on 81 *Vitis* genotypes to evaluate their antifungal resistance, ROS response, jasmonic acid (JA) levels, and changes in the antioxidant system following Bc inoculation [49]. The researchers discovered that resistant genotypes exhibited higher antioxidant enzyme activity and reduced ROS accumulation during Bc infection. Therefore, further investigation is necessary to confirm whether linalool effectively induces disease resistance by activating the antioxidant system in tomato fruits.

In the co-culture of B418 and T11-W, a significant up-regulation of the dimethyl disulfide and dimethyl trisulfide contents was observed among the VOCs produced. Previous studies have demonstrated that these compounds have notable inhibitory effects on the growth of phytopathogenic fungi, such as *Rhizoctonia solani* and Foc, while also promoting the growth of plant growth-promoting rhizobacteria (PGPR) [50,51]. Zuo et al. further showed that dimethyl trisulfide had a significant inhibitory effect on the growth and development of *F. acuminatum* [52]. Additionally, it exhibited significant inhibitory effects on *Penicillium italicum*, which infests citrus fruits, suggesting its potential as an effective option for controlling fruit diseases using fumigants in the future [53]. Sulfur-containing VOCs with antimicrobial activity can be considered as natural fumigants due to their reliable effectiveness in controlling plant pathogenic fungi and *Caenorhabditis elegans* [54]. Although the present study demonstrated the antimicrobial efficacy of sulfur-containing compounds, there remains a lack of clarity regarding their exact mode of action and potential synergistic effects. The biological activity of allicin, a sulfur-containing compound found in garlic (*Allium sativum* L.), has been shown to inhibit the growth of pathogenic microorganisms by reacting with proteins containing the -SH group in these microorganisms, mainly through the S-(O)-S moiety to form a mixed disulfide [55]. Additionally, it has been suggested that allicin may also alter the permeability of cell membranes, thereby disrupting the structural integrity of the organism and ultimately leading to cell death [56]. Therefore, we hypothesize that the increased antimicrobial effect may be attributed to the ability of dimethyl disulfide or trimethyl disulfide to penetrate the cell membranes of Bc and Foc, where they react with the sulfhydryl groups of peptidoglycan synthase. This direct interaction with the peptidoglycan in the fungal cell wall causes it to become thinner or develop voids, leading to an efflux of the contents and ultimately resistance. This hypothesis regarding membrane damage and induced systemic resistance in host plants was supported by previous research findings [57].

Benzene compounds, such as acetophenone and 2-phenylethanol, are an important class of organic compounds produced from the co-culture of B418 and T11-W. These compounds possess unique resonance stability and higher stability due to the presence of a benzene ring structure. Acetophenone is commonly found in plants and microorganisms as a natural product and has garnered attention for its structural simplicity, ease of modification, excellent bacteriostatic effect, and promotion of plant growth and development [58]. Rajabi et al. found that acetophenone and its derivatives have antimicrobial effects on fungi and Gram-positive bacteria, with significant inhibitory effects on *Mycobacterium tuberculosis* [59]. Tran et al. isolated two prenylated acetophenones from the Australian endemic plant *Acronychia crassipetala* and assessed their antibacterial activity against *Staphylococcus aureus* and *Entercoccus faecium* at low concentrations [60]. Furthermore, previous studies have demonstrated that 2-phenylethanol can control fruit pathogenic fungi, such as *Phytophthora infestans*, *B. cinerea*, and *Penicillium* sp., playing a crucial role in maintaining the freshness of fruits and vegetables [61]. Monggoot et al. suggested that 2-phenylethanol exhibits potent antimicrobial activity by disrupting cell membrane integrity while decreasing toxin content [62]. Zou et al., meanwhile, discovered that 2-phenylethanol effectively maintains fruit and vegetable freshness by damaging cell membranes and inducing ROS stress response to inhibit *B. cinerea* growth [63]. Therefore, both 2-phenylethanolandacetophenones hold great potential for future applications in biocontrol and play an essential role in the sustainable development of agriculture.

In summary, co-culture is an ecologically driven approach that can induce previously unexpressed biosynthetic pathways and enhance the metabolic capacity of chemically prolific microorganisms. This study provides evidence that co-culturing biocontrol strains can significantly enhance antagonistic activity against phytopathogenic fungi, primarily by stimulating the production of more volatile metabolites.

## 4. Materials and Methods

### 4.1. Strain, Medium, and Culture Conditions

The biocontrol strains *B. vietnamiensis* B418 (China General Microbiological Culture Collection Center, CGMCC No. 1212) and *T. harzianum* T11-W (CGMCC No. 7938) were isolated and preserved by the Environmental Microbiology Laboratory, Ecology Institute of Shandong Academy of Sciences. Fungal phytopathogens *Botrytis cinerea* (Bc) and *Fusarium oxysporum* f. sp. *cucumerium* Owen (Foc) were used as target pathogens for the examination of antagonistic activity.

### 4.2. Preparation of Bacterial and Spore Suspensions

*B. vietnamiensis* B418 was cultivated in TY-A medium (peptone 10 g L^−1^, yeast powder 1 g L^−1^, CaCl_2_ 0.2 g L^−1^, agar 15 g L^−1^, distilled water 1000 mL, pH 7.2–7.4) within a constant temperature incubator at 30 °C for 2 days. Subsequently, single colonies of B418 were picked and transferred into TY-L broth (peptone 10 g L^−1^, yeast powder 1 g L^−1^, CaCl_2_ 0.2 g L^−1^, distilled water 1000 mL, pH 7.2–7.4) and cultured with shaking at 30 °C on a shaker at 180 rpm for 24 h. After enumeration using a hematology counting plate, it was diluted and adjusted to 2 × 10^8^ cfu/mL with sterile distilled water (SDW).

*T. harzianum* T11-W was cultivated on PDA at 28 °C for 7 days until sporulation occurred. The conidia were suspended in SDW and enumerated on hematocrit plates to prepare a suspension of *Trichoderma* spores with a conidial content of 2 × 10^8^ spores/mL.

*B. vietnamiensis* B418 and *T. harzianum* T11-W suspensions were inoculated into 200 mL of MKB medium (modified King’s B broth; casein amino acids 20 g L^−1^, glycerol 10 mL, K_2_HPO_4_ 1.5 g L^−1^, MgSO_4_·7H_2_O 1.5 g L^−1^, distilled water 1 L, pH 7.2 ± 0.2) at 1% inoculum for the mono- and co-cultures. The cultures were cultivated at 28 °C and 180 rpm for 14 d. The fermentation broths of B418 mono-culture, T11-W mono-culture, and B418+T11-W co-culture (designated as B1, T11, and BT11, respectively) were collected by centrifugation at 12,000× *g* at 4 °C for 5 min. The fermentation filtrates were obtained by the filtration of fermentation broths through a 0.22 μm membrane filter (MF-Millipore, Merck, Darmstadt, Germany).

### 4.3. Scanning Electron Microscope (SEM)

The microstructure of B418 and T11-W in mono- and co-culture modes were examined using the Regulus SU8100 (Hitachi, Tokyo, Japan) according to the method described by Kaewmanee et al. [64], with slight modifications. Briefly, the cultures were fixed in 2.5% glutaraldehyde (*w*/*v*) at room temperature for 12 h, rinsed with 0.1 M phosphate buffer (pH 7.0) thrice for 15 min, and then washed with an ethanol gradient (30%, 50%, 70%, 90%, and 100% (*v*/*v*)) for 10 min to dehydrate. Finally, the samples were critically dried with carbon dioxide, mounted on aluminum stubs with conductive carbon cement, and coated with palladium by magnetron sputtering.

### 4.4. Determination of Antagonistic Effect

The bioactivity of VOCs produced by B418 and T11-W in mono- and co-culture modes against phytopathogenic fungi was assessed by inhibitory rates. The inhibition rate was determined following a previously described method [65] with minor modifications. Briefly, the spore suspension or mycelium collected in mono- and co-culture modes was spread evenly on the PDA, and a 6 mm diameter cover with a pathogen mycelium agar plug was inoculated at the center of the new PDA. Immediately after removing the lid, the two uncovered plates were docked and sealed with a double layer of cling film (a pathogen-inoculated plate was used as the control). It should be noted that the plate containing phytopathogens needs to be placed above the plate containing the fermentation broth. All the plates were incubated at 28 °C in a thermostat. The plates inoculated with Foc were incubated for 4 d, and the plates inoculated with Bc were incubated for 6 d. The colony diameter was measured using the criss-cross method [66], with six replicates set up for each treatment, and each experiment was conducted with three replicates.

The percentage of mycelial growth inhibition was calculated according to the following formula:

(1) $$Inhibition\;of\;mycelial\;growth\left(\%\right)=\frac{Rc-Rt}{Rc}\times 100\%$$

The variable Rc represents the diameter of the control colonies, while Rt denotes the diameter of the treatment colonies.

### 4.5. Verification of Antagonistic Effect

In this experiment, a three-compartment Petri dish was selected as the experimental vessel. Two experimental schemes were designed: (1) The bio-prophylaxis bacteria in the first compartment (PDA or TY-A) were accessed, and a cake of phytopathogens with a size of about 6 mm was inoculated in the center of the second compartment (PDA). The third compartment was left empty. (2) Activated carbon was added to the third compartment for adsorption of VOCs produced by the biocontrol agents. Subsequently, it was sealed with a sealing film and incubated at 28 °C in a constant temperature incubator. The growth of phytopathogens was measured every 24 h. Control plates were inoculated with phytopathogens only, without the addition of biocontrol bacteria and activated carbon. There were six replicates for each treatment, and the experiment was repeated three times.

### 4.6. Detached Fruit Experiment

This experiment selected tomato fruits with the same growth cycle and size. As listed in Table 1, there were 9 treatments designed for the experiment. Each tomato fruit was injected with 10 μL of pathogen spore suspension (1 × 10^3^ spores/mL), while the water control group was injected with 10 μL of SDW. Subsequently, all the tomato fruits were placed in sterile plastic boxes. After 6 h, PDA plates of mono- and co-cultures of B418 and T11-W were added to the plastic box (the control group was placed on PDA plates containing SDW). The plastic boxes were then sealed with a sealing film and placed in a constant temperature oven at 28 °C for incubation. The tomato fruits were observed every 12 h for 3 days. When the incidence rate of the fruits in the pathogen control group exceeded two-thirds, the control effects of the different treatment groups were counted. After counting, mycelia of the phytopathogens of the control and treatment groups were re-cultured on PDA plates to observe whether the VOCs would affect mycelial development. Each treatment had at least six biological replicates, and the experiment was repeated twice.

### 4.7. Collection, Identification, and Statistical Analysis of VOCs

GC-MS analysis was performed by Wekemo Tech Group Co., Ltd. (Shenzhen, China) as previously described [67]. B418 bacterial solution, T11-W spore suspension, and B418+T11-W co-culture bacterial solution were evenly spread in a headspace (HS) flask containing 20 mL of PDA medium. After incubating on the PDA medium for 5 d, the HS vials were tightly sealed using hole caps and PTFE/silicone septa. For GC-MS measurements, volatiles were extracted using a 50/30 μm DVB/CARonPDMS fiber for HS extraction, and chromatographic separation was carried out on an HP5-MS column (30 m × 0.25 mm × 0.25 μm; Agilent, Waldbronn, Germany). The automated extraction of volatiles was conducted using a CTC Trinity autosampler (Agilent, Waldbronn, Germany). Bacterial fluids and spore suspensions were incubated at 50 °C with shaking for 15 min. Volatiles from the headspace were extracted for 50 min by exposure to an SPME fiber with 50/30 μm DVB/CARonPDMS coating without agitation. The volatiles bound to the fiber were transferred to the GC-MS instrument and further analyzed on the Model 7890B gas chromatograph coupled to a Model Pegasus BT mass spectrometer.

The analytes were desorbed for 5 min in a split/splitless injector operating in splitless mode at 240 °C, equipped with a headspace glass liner (inner diameter of 1.5 mm, Xiangbo, China), and subsequently separated on DB-wax (30 m × 0.25 mm × 0.25 µm). Helium was used as the carrier gas at a constant flow rate of 1 mL/min. The oven program consisted of an initial temperature of 40 °C (held for 5 min), followed by an increase at a rate of 5 °C/min to reach 220 °C, and then further increased at a rate of 20 °C/min to reach 250 °C (held for 2.5 min). For mass spectrometry detection, electron impact ionization (EI) was employed with an energy level set at 70 eV, source temperature was set at 230 °C, quadrupole temperature was set at 150 °C, full-scan scanning mode was utilized, and mass range was scanned from 20 to 400 amu. Volatiles emitted by HB20111 were provisionally identified through computer searches guided by the National Institute of Standards and Technology (NIST) Mass Spectral Library2011 (NIST, Gaithersburg, MD, USA).

The KEGG database (https://www.genome.jp/kegg/pathway.html; URL (accessed on 30 April 2024)), HMDB database (https://hmdb.ca/metabolites; URL (accessed on 30 April 2024)), and LIPIDMaps database (http://www.lipidmaps.org/; URL (accessed on 30 April 2024)) were used to annotate the identified metabolites.

### 4.8. Determination of Antagonistic Activity of VOC Standard

Based on the results of the metabolomics analysis, six compounds were identified that exhibited significant elevation in the co-culture mode. These compounds included dimethyl disulfide, dimethyl trisulfide, linalool, acetophenone, geranone, and 2-phenylethanol.

A plug of phytopathogens measuring approximately 6 mm in size was inoculated in the center of a Petri dish containing PDA medium. Subsequently, a sterile piece of filter paper with a radius of 2 cm was placed in the center of the lid of the cup and a drop of standard solution (at a concentration of 1 × 10^−5^ mg/L) was added. The dish was quickly covered and sealed with a film to prevent evaporation. The plates were then incubated at a constant temperature of 28 °C, and the growth of phytopathogens was monitored every 24 h. A negative control with drops of SDW and a solvent-only control with drops of DMSO, which was the solvent used for the dissolving of the VOC standards, were used as controls. Each treatment had at least six biological replicates, and this experiment was repeated twice for validation purposes.

### 4.9. Statistics

The data were statistically analyzed using SPSS 26.0 (SPSS Inc., Chicago, IL, USA) and PRISM 8.3.0.538 software (GraphPad Software Inc., San Diego, CA, USA). The difference among the treatments was determined using a one-way analysis of variance (ANOVA). A Duncan’s multiple range test was applied to determine the significant difference (*p* < 0.05).

## 5. Conclusions

Co-cultivation of biocontrol strains is an effective strategy to enhance antagonistic activity against phytopathogenic fungi by stimulating the production of more VOCs. In this study, we demonstrated that co-cultivation of *Burkholderia vietnamiensis* B418 and *Trichoderma harzianum* T11-W improved inhibitory activity against *Botrytis cinerea* and *Fusarium oxysporum* f. sp. *cucumerium* Owen, showing good control on both plates and tomato fruits. The results of the GC-MS analysis revealed that under the co-culture conditions of B418 and T11-W, several volatile compounds, including linalool, dimethyl trisulfide, dimethyl disulfide, geranylacetone, 2-phenylethanol, and acetophenone exhibited strong inhibitory effects compared with those from the individual cultures. A significant increase in the content of several VOCs with antimicrobial effects was observed. The enhanced antagonistic activity against fungal phytopathogens in co-culture can be attributed to the increased content of these compounds. However, it should be noted that this study did not investigate the effects of VOCs on human and food safety. Further research is needed to explore the potential synergistic effects between different VOCs.

## Figures and Tables

**Table 1:** Experimental designs for detached fruit experiments.

No.	Name	Description
1	CK (1)	Fruits were injected with sterile distilled water and boxed into freshwater plates
2	CK (2)	Fruits were injected with *Botrytis cinerea* (Bc) spore solution and boxes were placed in freshwater plates
3	CK (3)	Fruits were injected with *Fusarium oxysporum* f. sp. *cucumerium* Owen (Foc) spore solution, and the box was placed in freshwater plates
4	B1 (1)	Fruits were injected with Bc and boxed into B418 plates
5	B1 (2)	Fruits were injected with Foc and boxed into B418 plates
6	T11 (1)	Fruits were injected with Bc and boxed into T11-W plates
7	T11 (2)	Fruits were injected with Foc and boxed into T11-W plates
8	BT11 (1)	Fruits were injected with Bc and boxed into B418+T11-W plates
9	BT11 (2)	Fruits were injected with Foc and boxed into B418+T11-W plates

## Data Availability

The data that support the findings of this study are available from the corresponding author upon reasonable request.
